# Uncovering Genomic Features and Maternal Origin of Korean Native Chicken by Whole Genome Sequencing

**DOI:** 10.1371/journal.pone.0114763

**Published:** 2014-12-11

**Authors:** Woori Kwak, Ki-Duk Song, Jae-Don Oh, Kang-Nyeong Heo, Jun-Heon Lee, Woon Kyu Lee, Sook Hee Yoon, Heebal Kim, Seoae Cho, Hak-Kyo Lee

**Affiliations:** 1 C & K genomics, Seoul, Republic of Korea; 2 Interdisciplinary Program in Bioinformatics, Seoul National University, Seoul, Republic of Korea; 3 Genomic Informatics Center, Hankyong National University, Anseong, Republic of Korea; 4 Poultry Science Division, National Institute of Animal Science, Cheonan, Republic of Korea; 5 Department of Animal Science and Biotechnology, Chungnam National University, Daejeon, Republic of Korea; 6 Inha Research Institute for Medical Sciences, Inha University School of Medicine, Inchon, Republic of Korea; 7 Department of Agricultural Biotechnology and Research Institute for Agriculture and Life Sciences, Seoul National University, Seoul, Republic of Korea; BiK-F Biodiversity and Climate Research Center, Germany

## Abstract

The Korean Native Chicken (KNC) is an important endemic biological resource in Korea. While numerous studies have been conducted exploring this breed, none have used next-generation sequencing to identify its specific genomic features. We sequenced five strains of KNC and identified 10.9 million SNVs and 1.3 million InDels. Through the analysis, we found that the highly variable region common to all 5 strains had genes like *PCHD15*, *CISD1*, *PIK3C2A*, and *NUCB2* that might be related to the phenotypic traits of the chicken such as auditory sense, growth rate and egg traits. In addition, we assembled unaligned reads that could not be mapped to the reference genome. By assembling the unaligned reads, we were able to present genomic sequences characteristic to the KNC. Based on this, we also identified genes related to the olfactory receptors and antigen that are common to all 5 strains. Finally, through the reconstructed mitochondrial genome sequences, we performed phylogenomic analysis and elucidated the maternal origin of the artificially restored KNC. Our results revealed that the KNC has multiple maternal origins which are in agreement with Korea's history of chicken breed imports. The results presented here provide a valuable basis for future research on genomic features of KNC and further understanding of KNC's origin.

## Introduction

While Korean Native Chicken (KNC) have a relatively slow growth rate compared to commercial broiler breeds, the breed is preferred in Korea for consumption due to its low-fat and high-protein meat content and taste[1]. KNC is an artificially restored breeds and the its history can be found in the national report of Food and Agriculture Organization of the United Nations (FAO) (FAO website, Available: ftp://ftp.fao.org/docrep/fao/010/a1250e/annexes/CountryReports/KoreanRepublic.pdf. Acessed 2014 June 4). According to the report, it is believed that KNC came from China or Southeast Asia about 2000 years ago. In early 20^th^ century, South Korea was blighted with several wars like Second World War and Korean War. The genetic resource of KNC was greatly reduced during this time period. In addition, during the Japanese occupation and post-war recovery period, a number of foreign breeds were imported, further pushing KNC to extinction. To remedy the loss of endemic genetic resources, the National Institute of Animal Science, Rural Development Administration of Korea, implemented a project to restore KNC in the late 1970s. Researchers collected KNCs remained in the countryside farms and increased its population size. In addition to the effort of research institute, general farm also showed interest in KNC and many KNCs were raised in the private sector. However, the absence of standard breeding concept brought lack of uniformity and productivity of KNCs and there was a need to form standard population. In 1992, research team of national institute, university and industry researchers developed and restored the standard KNC at the end of 8^th^ generation. The restored KNC breed is composed of five strains defined by its plumage color: red, yellow, black, grey, and white.

Numerous studies have been carried out to understand the origin and unique genomic characteristics of KNC. Kong et al. studied the genetic variants between KNC and other chicken breeds using 15 chicken specific microsatellite markers [2]. Oh et al. identified SNPs within the uncoupling protein gene and characterized the association between these SNPs and daily percent lay of KNC[3]. Hoque et al. analyzed the D-loop sequences of the mitochondrial DNA in KNC to differentiate between strains and conducted phylogenetic analysis [4], [5]. In these studies, they showed that KNCs were derived from China with multiple origins.

Although next generation sequencing is widely used for genomic studies in domestic animals, there is a paucity of studies on KNC, especially on variants in the whole genome and the origin of KNC using whole mitochondrial genome. In the present study, we presented results of the first whole genome sequencing of KNC, using 5 strains. We sought to identify genomic variants through re-sequencing, *de novo* assembly and gene prediction of the KNC genome, and investigation of genetic differences by comparison with the reference genome. In addition, we determined the whole mitochondrial genome sequences to construct a phylogenetic tree to investigate the maternal origin of the restored KNC by integrating them with sequences from a previous study on chicken domestication [6] and published chicken whole mitochondrial genome sequences. From these analyses, we identified the characteristics of the KNC genome at the single nucleotide level and isolated the maternal origin of artificially restored KNC breed.

## Materials and Methods

### Ethics Statement

All animal care and experimental procedures were reviewed and approved by the Institutional Animal Care and Use Committee of the National Institute of Animal Science (No. 2012-C-037).

### Sample collection

Whole Blood samples (1ml) were collected respectively from five male Korean Native Chickens, each with a different plumage color: red (KNC_R), yellow (KNC_Y), white (KNC_W), black (KNC_L) and grey (KNC_G). Blood drawn from each sample was treated with heparin to prevent clotting.

### Library preparation and read generation

DNA(20ug) was isolated from whole blood using G-DEXTMIIb Genomic DNA Extraction Kit(iNtRoN Biotechnology, Seoul, Korea) according to the manufacturer's protocol. We randomly sheared genomic DNA using Covaris System to generate inserts of ∼300bp. A genomic DNA library was constructed for each sample using TruSeqTM DNA Sample Preparation Kit v2 (Illumina, San Diego, CA, USA) following the manufacturer's protocol. To generate the raw data for each sample, 101 cycle paired-end sequencing using Hiseq 2000 was carried out. The quality of the whole genome sequencing reads from all samples was checked using FastQC [7] and rawdata quality control process was conducted using Trimmomatic[8] with the parameter(ILLUMINACIP:TruSeq3-PE-2.fa:2:30:10, MINLEN:75). Generated sequencing data and quality control result are summarized in Table 1. Reads retained after these basic quality control process were used for further analysis.

**Table 1:** Summary of sequencing information and rawdata QC result using trimmomatic.

	Number of Reads	Read Length	Coverage(X)	GC(%)	Calculated Insert Size	Remained Read after Trimmomatic
KNC16	350,530,416	101	33.8	40	277	349,573,474 (99.73%)
KNC24	369,509,968	101	35.6	40	268	368,715,114 (99.78%)
KNC3	367,190,708	101	35.4	40	271	366,406,916 (99.79%)
KNC40	354,548,474	101	34.2	40	256	353,489,760 (99.70%)
KNC53	375,424,656	101	36.2	40	272	374,355,934 (99.72%)

Coverage was calculated based on the size of reference genome Galgal4(1046.93MB).

### Variant calling

Paired-end reads of each sample were mapped to the chicken reference genome (Gallus_gallus 4.0/galGal4) from the Ensembl database(ver.74) using Bowtie2 [9] v2.2.3 with the default options. Using the “REMOVE_DUPLICATES = true” option in “MarkDuplicates” command-line tool of Picard (http://picard.sourceforge.net, v.1.109), potential PCR duplicates were removed. We then used SAMtools [10] to create index files for reference and bam files. Genome Analysis Toolkit (GATK) [11] was used for downstream processing and variant calling. Local realignment was conducted using GATK to correct misalignments due to the presence of indels. The “UnifiedGenotyper” of GATK was used for calling candidate single nucleotide variants (SNVs) and InDel. To avoid possible false positive variants, argument “VariantFiltration” of the same software was adopted with the following options: 1) SNVs with a phred-scaled quality score of less than 30 were filtered; 2) SNVs with QD(unfiltered depth of non-reference samples; low scores are indicative of false positives and artifacts) <5 were filtered; 3) SNVs with FS (phred-scaled P value using Fisher's exact test)>200 were filtered as FS represents variation on either the forward or the reverse strand, which are indicative of false-positive calls; 4) SNVs with MQ0(the number of reads which have mapping quality zero)>4 or MQ0/DP(proportion of mapping quality zero reads over total depth)>0.1 were filtered to remove uncertain calls; 5) more than 3 SNVs within 10bp window were filtered. Likewise, we also excluded variants that were predicted to overlap with InDel mutation, or overlap with zero coverage regions in more than one sample. Variant density was calculated using VCFtools[12].

### Genome assembly, alignment and gene prediction

Sequencing errors were discarded using error correction module of Allpaths-LG [13]. Fq2fa was used to merge the error corrected paired end reads of each sample to a single shuffled form fasta file with the filter option for filtering N bases in the reads. We assembled the error corrected paired-end reads using IDBA_UD [14] with the pre_correction option and mink value 31. All other options were set to default. Gaps (N bases) in the assembled sequences were filled using Gapcloser [15] with a parameter k value of 31.

Unaligned reads from Bowtie2 with no-mixed option were remapped to the assembled sequences to detect unique sequences for each KNC strain. Sequences with a minimum length of 2000 bp and an average mapping read depth of over 10 were used for gene prediction.

Before gene prediction for the filtered sequence, RepeatMasker [16] was run with the following options: species chicken, nolow, and no_is. AUGUSTUS [17] was run across repeat masked sequences for gene prediction with species = chicken option. The protein sequences from results of augustus were parsed using in-house python script. Amino acid sequences over 31bp, the shortest sequence length of annotated Galgal4 protein sequence, used as input for Blastp [18] search with Galgal4 Ensembl(ver.74) protein sequence database. The result of the blast search was filtered by subject and query coverage (over 90%).

The overall processes of genome assembly and gene prediction results are shown in Figure S1 in S1 File.

### Phylogenetic tree analysis

Whole mitochondria genome sequences of five KNC strains were constructed using variants from each sample based on the sequence of the chicken mitochondria reference genome (NC_001323). To construct a phylogenetic tree, 61 whole mitochondrial genome sequences from the chicken domestication study[6] and 9 sequences from NCBI database were used in conjunction with the sequences of the five KNC. Detailed information about the 75 whole mitochondria genome sequences used in this study is summarized in Table S1 in S1 File. Multiple sequence alignment was conducted using PRANK[19]. Two large gaps in the D-loop region and gaps generated by one specific sample were manually removed using Bioedit[20]. The final alignment was composed of 16,788bp nucleotide positions divided into 39 partitions including individual genes and noncoding regions.

The methods of Bayesian approach and neighbor joining were used to build the phylogenetic trees. Modeltest v3.7 [21] was carried out to determine a proper evolution model for Bayesian approach using MrBayes v3.2.2 [22]. The results of the likelihood scores for each of the 56 models using PAUP[23] were used as input data for Modeltest. The results of Modeltest based on AIC(Akaike Information Criterion) value were showed in Table S2 in S1 File. The phylogenetic tree using MrBayes was built with the following options: nst = 6, rates = gamma and ngen = 2,000,000. The PHYLIP package [24] was used to construct a consensus phylogenetic tree using neighbor joining method with bootstrapping. The Ts/Tv ratio was estimated using the kimura-2-parameter evolution model in TREE-PUZZLE [25]. An overview of our phylogenetic analysis is shown in Figure S2 in S1 File.

## Results

### Variant Calling

**Figure 1:**
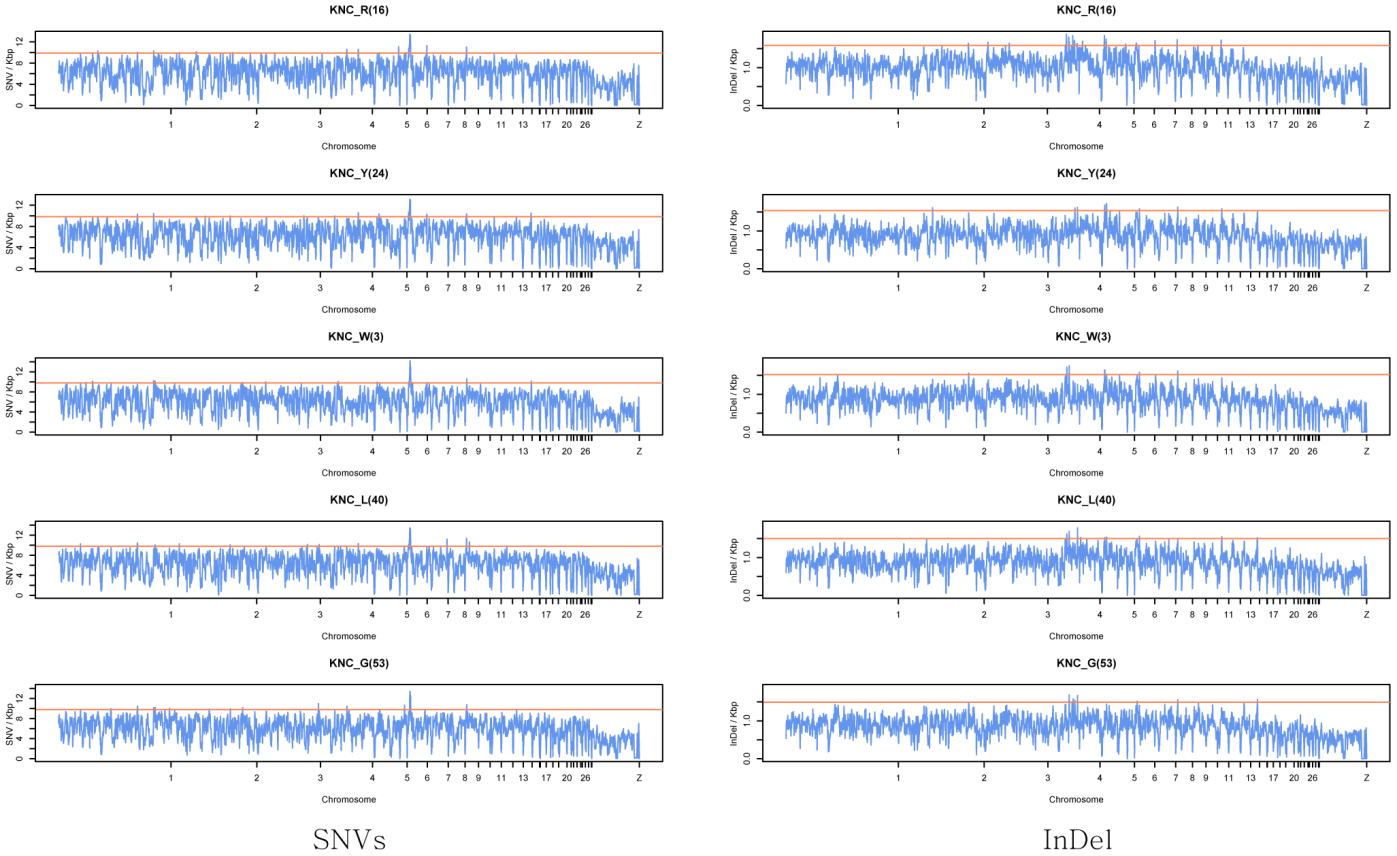
Manhattan plot showing the rate of SNVs and InDels per 500kbp window for the autosomes and sex chromosome. The red line denotes the top 1% of empirical distribution.

The overall average mapping rate for the five chicken samples was 97.52% and the percentage of reads with one concordant mapping to the reference genome was 87.43% (Table S3 in S1 File). Mean coverage depth was 35.04 and the average calculated insert size was 268.8 (Figure S3 in S1 File). After variant calling and filtering, 1.3 million InDels and 10.9 million SNVs were retained. Of the 10.9 million SNVs identified in this study, 6.3 million SNVs have previously been reported (57.62%) while 4.6 million SNVs were novel variants (42.37%) based on Ensembl database ver.74. Figure S4 in S1 File shows the distribution of both novel and previously reported SNVs in each chromosome. The number of strain specific variants is summarized in Table S4 in S1 File. KNC_L(40) had a higher number of strain specific variants than the other four KNC strains. The proportion of strain specific SNVs made up 28% of all identified SNVs and 21% of the total identified SNVs were common to all strains (Figure S5 in S1 File). For InDel variants, 20% of the identified INDELs were strain specific while 30% were common to all five KNC strains (Figure S6 in S1 File). The ratios of the number of chromosomal variants to the total number of variants are displayed in Figure S7 in S1 File with the chromosomal length ratio distribution. The ratio of variants showed similar distribution patterns with the ratio of chromosome length. Most of the SNVs and InDels were located in the intergenic region and the intron region had the next highest proportion of variants (Figure S8 in S1 File). The number of SNVs and InDels per 500kbp window was calculated for each chromosome and the results are represented in Fig. 1. The top 1% of mutation rates was used to distinguish the significant peaks on the SNP and InDel distributions (the top 1% is marked off with a red line). In the plot, SNV rates showed peak points on chromosome 6 and 9, in all five strains and the overall distributions of InDel rates showed peak points in chromosome 5, 6, and 8. There were 25 and 16 genes in the highly variable SNVs and InDels windows where to be in common to all five KNC strains. The name of genes and number of variants in each gene were summarized in Table 2. The positions of synonymous and non-synonymous variants in each gene are summarized in Table S5 in S1 File.

**Table 2:** List of genes and the number of variants in the highly variable regions of SNVs and InDels.

SNV_CHR	Genes	Number of Variants	Indel_CHR	Genes	Number of Variants
6	*RASGEF1A*	769	5	*PLEKHA7*	184
6	ENSGALG00000027731	6	5	*RPS13*	9
6	*CSGALNACT2*	269	5	*PIK3C2A*	155
6	*RET*	1,467	5	*NUCB2*	62
6	*BMS1*	417	5	*KCNJ11*	0
6	*C10ORF57*	127	5	*ABCC8*	151
6	ENSGALG00000002638	646	5	*USH1C*	131
6	ENSGALG00000024441	269	5	*OTOG*	266
6	*ANXA11*	443	6	*RASGEF1A*	65
6	*ECD*	185	6	ENSGALG00000027731	2
6	*FAM149B1*	236	6	*CSGALNACT2*	38
6	*DNAJC9*	41	6	*RET*	159
6	*TFAM*	140	8	*CDC73*	218
6	*UBE2D1*	411	8	*B3GALT2*	0
6	*CISD1*	171	8	*TROVE2*	41
6	*IPMK*	172	8	*UCHL5*	54
6	ENSGALG00000024315	10			
6	*PCHD15*	6,625			
9	ENSGALG00000006724	7,831			
9	5S_rRNA	5			
9	ENSGALG00000027551	0			
9	*IMP4*	679			
9	*CCDC61*	48			
9	ENSGALG00000006716	73			
9	*VPS8*	1,098			

The number of variants was based on the result of multi-sample calling from GATK.

### Unaligned read assembly and gene prediction

Read assembly for each strain was filtered by a minimum sequences length of 2,000bp. This resulted in an average of 60,014.8 sequences with an average sequence length of 15,965.6 bp. The average N50 length was 27,009.2 bp and KNC_W (3) assembled into the longest sequence among the five samples with a length of 491,386 bp. The average sum of the total assembled sequences was 957 Mbp, which covered about 91.4% of the 1,046.93 Mb of the *Gallus gallus* 4.0 reference genome. Through the gapclose step conducted after assembly, N within the assembled sequence was filled. On average, 74,913.2 N bases were filled through this step leaving an average of 42,081 N bases after the step. The average GC contents of the assembled sequences were about 41.0% which is about 1.2% lower than the GC ratio of the reference genome at 42.2%. The details of the assembly for each strain are described in Table S6 in S1 File.

To find assembled sequences that are distinct from the reference genome, unaligned reads were separately aligned to the assembled sequences of each strain. On average, about 26% of the unaligned reads could be aligned to the assembled sequences and the details of the alignment using Bowtie2 is described in Table S7 in S1 File. For the sequences to which the unaligned reads were aligned to, sequences with the average mapped base depth coverage of less than 10X were filtered. This resulted in 1897, 2310, 2399, 2215 and 2127 sequences for KNC_R (16), KNC_Y(24), KNC_W(3), KNC_L(40), and KNC_G(53), respectively. After conducting gene prediction using filtered sequences, the following number of predicted genes were found for each strain: 463 for KNC_R(16), 584 for KNC_Y(24), 683 for KNC_W(3), 557 for KNC_L(40) and 609 for KNC_G(53). Among these, 28 genes were found in common between the five strains. After filtering the uncharacterized proteins, 14 genes were remained (Table 3).

**Table 3:** List of commonly predicted genes from assembled sequences of each sample.

Ensembl Gene ID	Gene Symbol	Description
ENSGALG00000000155	*OR6A2*	olfactory receptor, family 6, subfamily A, member 2
ENSGALG00000003613	*COR4*	Gallus gallus olfactory receptor 4 (COR4)
ENSGALG00000004411	*OR5I1*	olfactory receptor, family 5, subfamily I, member 1
ENSGALG00000016152	*LY6E*	Gallus gallus lymphocyte antigen 6 complex, locus E (LY6E)
ENSGALG00000016153	*PSCA*	prostate stem cell antigen
ENSGALG00000017348	*COR3'BETA*	Gallus gallus olfactory receptor, family 51, subfamily M, member 1 (OR51M1)
ENSGALG00000025840	*COR9*	Olfactory receptor-like protein COR9
ENSGALG00000025885	*COR7A*	Gallus gallus chick olfactory receptor 7a (COR7A)
ENSGALG00000026025	*OR8D4*	Gallus gallus olfactory receptor, family 8, subfamily D, member 4 (OR8D4)
ENSGALG00000026035	*COR6*	Gallus gallus chick olfactory receptor 6 (COR6)
ENSGALG00000026475	*SLURP1*	secreted LY6/PLAUR domain containing 1
ENSGALG00000026595	*COR3*	Gallus gallus olfactory receptor, family 8, subfamily U, member 1 (OR8U1)
ENSGALG00000027094	*OR11A1*	olfactory receptor, family 11, subfamily A, member 1
ENSGALG00000028893	*OR5AS1*	olfactory receptor, family 5, subfamily AS, member 1

### Phylogenomic analysis

**Figure 2:**
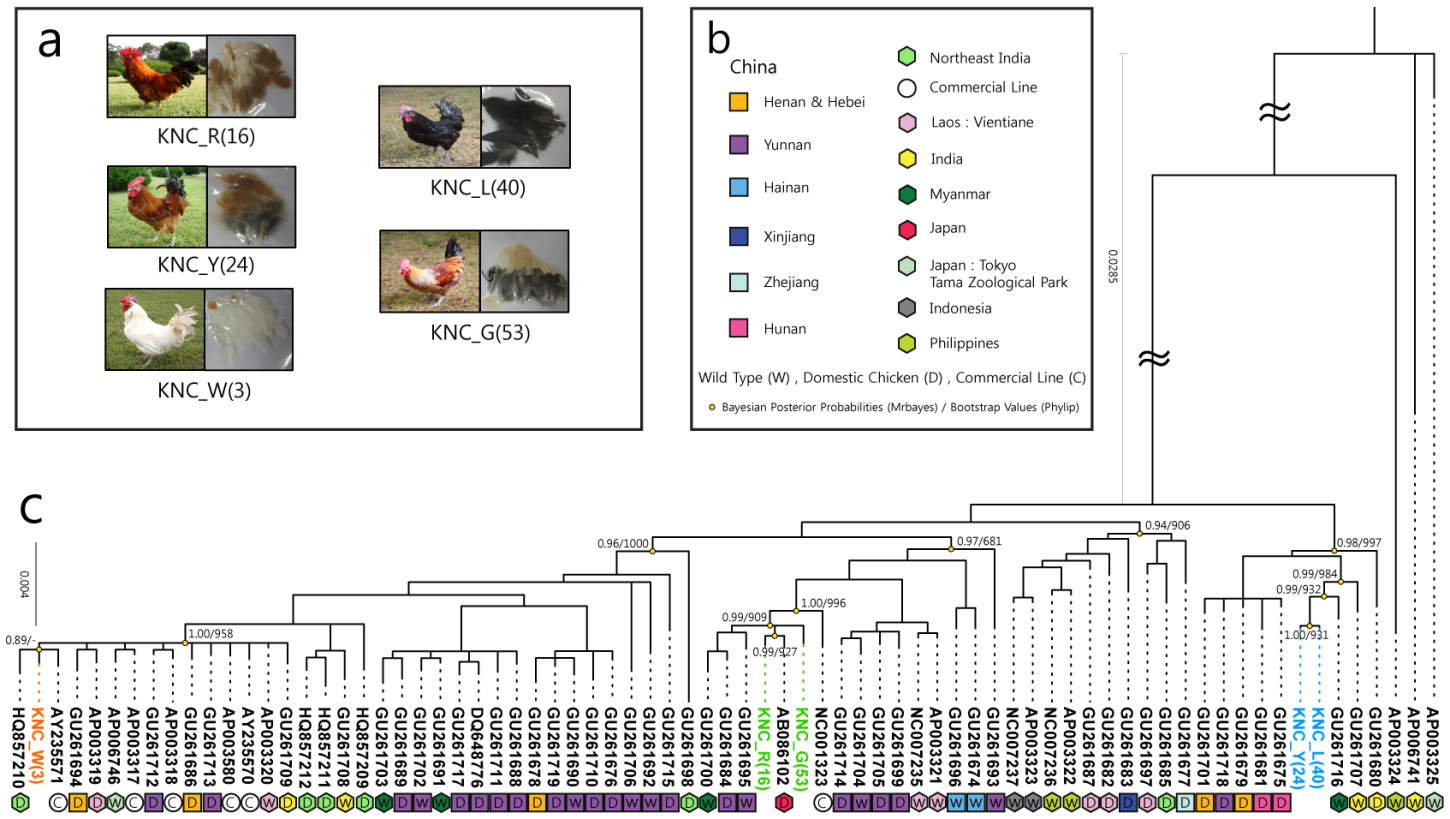
Phylogenetic tree using 75 whole MT genome sequences, including five Korean Native Chickens from this study. Box a shows the morphology and plumage color of the different KNC strains. Box b shows the legend (sample type and locality) for the phylogenetic tree in c. In the phylogentic tree, values (Bayesian posterior probability <0.8 or bootstrap value <600) were marked with a “-“ symbol.

A phylogenetic tree was constructed using MrBayes based on Bayesian inferences with 75 whole mitochondrial genome sequences from commercial chicken breeds of China, Japan, Southeast Asia, South Asia, and the KNCs (Fig. 2). The constructed tree showed that the 75 OTUs were broadly divided into five groups. Although the topology of the NJ tree (Figure S9 in S1 File) showed different ordering of groups, the branching pattern of each group was consistent with the tree constructed using Bayesian inference methods.

The KNC was divided into three different groups with KNC_Y(24) and KNC_L(40) forming one group, and KNC_G(53) and KNC_R(16) each forming separate groups. KNC_L(40) and KNC_Y(24) showed the closest phylogenetic relationship and their sister relationship OTU was GU261716, a red jungle fowl sampled in Myanmar. The OTU closest to KNC_R(16) was AB086102. This silky chicken sampled in Japan was closer to KNC_R(16) than KNC_G(53). Other OTUs clustering with KNC_G(52), KNC_R(16) and AB086102 was GU261695, GU261684 and GU261700. GU261695 and GU261684 area wild type and domestic chicken, respectively, sampled from the Yunnan region of China. Similar to GU261716, which had sister relationship with KNC_L(40) and KNC_Y(24), GU261700 is a wild type OTU sampled in Myanmar. The OTU closest to KNC_W(3) was AY235571 and HQ857210. AY235571 is a commercial breed and HQ857210 is a domestic chicken sampled in Northeast India. The sampling localities of the OTUs closest to the KNC groups were geographically varied. Taken together, the results showed that the five strains of KNC have multiple maternal origins.

## Discussion

### Genetic variants and specific genome sequences of KNC

This is the first whole genome sequencing study on KNC. 4.6million SNVs (42.37%) were novel variants based on the Ensembl database (ver.74). A whole genome sequencing study such as this one is beneficial as it provides more detailed information about genetic variants for local animal breeds than SNP chip analysis. Among common genes in highly variable regions of KNC, some genes had been previously studied for their association with phenotypic traits of chicken. *PCHD15*(protocadherin 15) is known to play an important role in the development of chicken cochlea[26]. It assists to form tip-link filament in sensory hair cells and the genetic variant of this gene can affect the mechanotransduction in sensory hair cell[27], [28]. *CISD1*, CDGSH Iron-Sulfur Domain-Containing Protein 1, also known as mitoNEET, is located in outer membrane of mitochondria and regulates oxidative capacity of mitochondria which in turn affects energy metabolism[29]. *PIK3C2A*(phosphatidylinositol-4-phosphate 3-kinase, catalytic subunit type 2 alpha) is influenced by miR-509-3p and it is known to be related with the growth inhibiting effect of miR-509-3p[30]. *NUCB2* protein has a calcium binding property and interacts with calcium during egg shell fabrication [31]. One of phosphopeptides extracted from eggshell proteins was estimated to be the hypothetical protein of *NUCB2* [32].

Commonly predicted genes identified through assembly of unaligned reads are likely to have large sequence differences to the reference genome than SNV or small InDels. The assembly of unaligned reads based on whole genome assembly could be an effective approach to identify specific genome sequences and its location in the genome, which is usually difficult to find using re-sequencing. *PSCA*, Prostate Stem Cell Antigen, is known to be related with auditory sense of chicken. It is highly expressed in telencephalon and peripheral ganglia of chicken and known to be correlated with expression of α7-containing nicotinic acetylcholine receptors (α7-nAChRs)[33]. And most of genes were olfactory receptor genes predicted in unaligned read assembly of 5 KNCs. Previous study about the location of structural variation in chickens showed that numerous CNVs were located in genes related with olfactory receptor genes[34]. Although the type of structural variants in this study was not expected as CNVs, structural variations such as insertion might be also related with the olfactory receptor gene repertoires of KNC.

This whole genome sequencing study using re-sequencing and unaligned read assembly provided more detailed information about the genomic features of KNC compared to the previous studies. However, the relationship between these features and KNC specific traits are still unclear. Additional studies are necessary to establish the relationship between KNC specific traits and genomic features identified in this study.

### Maternal origin of Korean Native Chicken

Phylogenetically, the closest neighbors to KNC in each group were a wild type sample from Myanmar(GU261716), a Japanese domestic chicken(AB086102) and a commercial line sample(AY235571). Same as Bayesian approach, GU26176, AB086102 and AY235581 were also closest neighbor of KNC in neighbor joining tree. However, there was little difference between the Bayesian posterior probability tree and the neighbor joining tree using bootstrapping. Bootstrap values of higher node of KNC that showed the reliability of tree topology were lower than Bayesian posterior probability and the tree topology was slightly different in clustering order of sample. Nevertheless, there was no difference in sample list in each group and we think this was important to identify the maternal origin of KNC.

Although one previous study was performed to determine the maternal origin of KNC using D-loop variation of mtDNA, no comparative phylogenomic analyses have been carried out with the whole mitochondrial genomes of various chicken breeds including neighboring countries as well as KNC. The result of comparative phylogenomic analyses was concordant with the previous study, showing that the five KNC strains had multiples origins. Moreover, our result indicated the possibility of another inflow route in addition to china. To some extent, these results are concordant to the historical records of KNC in Korea. KNC stock was supplemented with an improved Japanese breed due to a decline in productivity during the Japanese colonial rule in the early 1900s. Also, during the recovery period after the war in 1960s, Korea received fertilized eggs of a commercial breed from the ECA(Economic Cooperation Administration). These historic events support the tree topology and the closest neighbor of the KNC in the phylogenetic tree.

Our study is the first phylogenetic study about five restored KNC based on complete mitochondrial genome sequences. Our results expand the current knowledge about the origin of artificially restored KNC and acts as a basis for future conservation studies and optimization of breeding strategy.

## Supporting Information

S1 File **Supporting figures and tables.** Figure S1. Overall process of genome assembly and gene prediction. Table S1. List of GeneBank ID, Location and Type of 75 individuals used in phylogenomic analysis. Table S2. The result summary of Modeltest using 56 models from PAUP. Figure S2. An overview of the phylogenetic analysis process. Table S3. The result summary of read mapping using Bowtie2. Figure S3. Insert size distributions of 5 KNC samples. Figure S4. Distribution of known and novel SNVs in each chromosome. Table S4. The result summary of variant calling using GATK. Figure S5. Ratio of breed specific SNV and the proportion of each breed in breed specific SNVs. Figure S6. Ratio of breed specific InDel and the proportion of each breed in breed specific InDels. Figure S7. Ratio of the number of chromosomal variants to the total number of variant. Figure S8. Ratio of each variant location in three variant types (SNP, InDel and CNV). Table S5. The list of synonymous and non-synonymous variants in common genes of highly variable regions. Table S6. The result summary of Korean Native Chicken genome assembly using IDBA_UD. Table S7. The result summary of remapping unaligned reads to assembled genome using bowtie2. Figure S9. Neighbor joining tree of 75 samples used in this analysis.
(DOCX)
