# Supplementary material for: Uncovering Genomic Features and Maternal Origin of Korean Native Chicken by Whole Genome Sequencing
Source: PLoS One. 2014 Dec 11;9(12):e114763. doi: 10.1371/journal.pone.0114763 (PMC4263466; doi:10.1371/journal.pone.0114763)
Supplement: S1 File — Supporting figures and tables. Figure S1. Overall process of genome assembly and gene prediction. Table S1. List of GeneBank ID, Location and Type of 75 individuals used in phylogenomic analysis. Table S2. The result summary of Modeltest using 56 models from PAUP. Figure S2. An overview of the phylogenetic analysis process. Table S3. The result summary of read mapping using Bowtie2. Figure S3. Insert size distributions of 5 KNC samples. Figure S4. Distribution of known and novel SNVs in each chromosome. Table S4. The result summary of variant calling using GATK. Figure S5. Ratio of breed specific SNV and the proportion of each breed in breed specific SNVs. Figure S6. Ratio of breed specific InDel and the proportion of each breed in breed specific InDels. Figure S7. Ratio of the number of chromosomal variants to the total number of variant. Figure S8. Ratio of each variant location in three variant types (SNP, InDel and CNV). Table S5. The list of synonymous and non-synonymous variants in common genes of highly variable regions. Table S6. The result summary of Korean Native Chicken genome assembly using IDBA_UD. Table S7. The result summary of remapping unaligned reads to assembled genome using bowtie2. Figure S9. Neighbor joining tree of 75 samples used in this analysis. (DOCX) [file pone.0114763.s001.docx]

**<Supporting Information>**

**
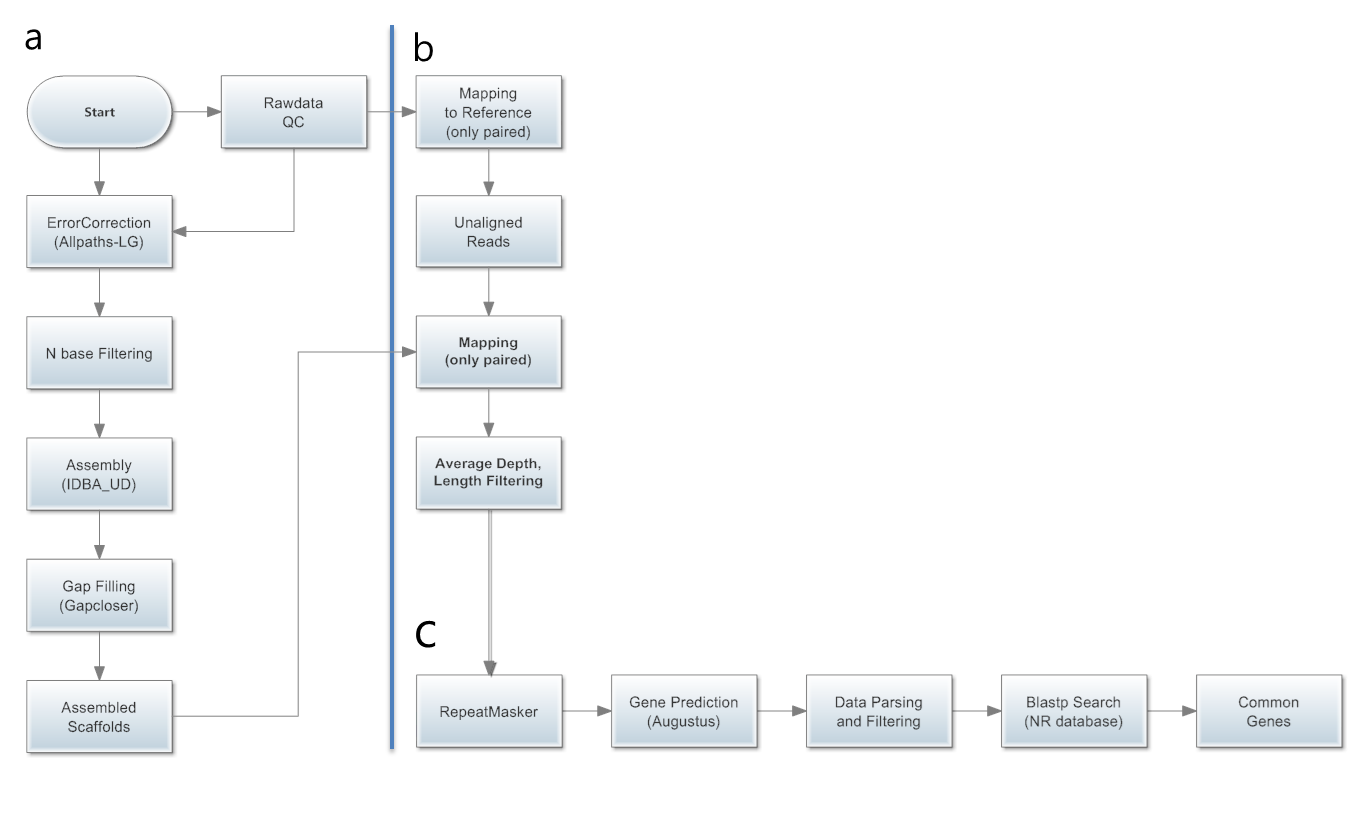
**

Figure S1. Overall process of genome assembly and gene prediction.

Table S1. List of GeneBank ID, Location and Type of 75 individuals used in phylogenomic analysis.

| **GenBank ID** | **Location** | **Type** |
| --- | --- | --- |
| AB086102 | Japan: Hiroshima | domestic chicken |
| GU261684 | China: Yunnan | domestic chicken |
| GU261695 | China: Yunnan | wild fowl |
| GU261700 | Myanmar | wild fowl |
| NC_007235 | Laos: Vientiane | wild fowl |
| GU261704 | China: Yunnan | wild fowl |
| GU261705 | China: Yunnan | domestic chicken |
| GU261714 | China: Yunnan | domestic chicken |
| GU261699 | China: Yunnan | domestic chicken |
| GU261674 | China: Hainan | wild fowl |
| GU261696 | China: Hainan | wild fowl |
| GU261693 | China: Yunnan | wild fowl |
| GU261701 | China: Henan | domestic chicken |
| GU261675 | China: Hunan | domestic chicken |
| GU261681 | China: Hunan | domestic chicken |
| GU261718 | China: Yunnan | domestic chicken |
| GU261679 | China: Henan | domestic chicken |
| GU261680 | Southern India | domestic chicken |
| GU261716 | Myanmar | wild fowl |
| GU261707 | India | wild fowl |
| NC_007236 | Philippine: Manila | wild fowl |
| NC_007237 | Indonesia: Bali | wild fowl |
| GU261687 | Laos | domestic chicken |
| GU261682 | Laos | domestic chicken |
| GU261683 | China: Xinjiang | domestic chicken |
| GU261677 | China: Zhejiang | domestic chicken |
| GU261697 | Southern India | domestic chicken |
| GU261685 | Northeast India | domestic chicken |
| GU261686 | China: Henan | domestic chicken |
| GU261713 | China: Yunnan | domestic chicken |
| AP003317 | Commercial Line | domestic chicken |
| AY235571 | Commercial Lines | domestic chicken |
| AP003318 | Commercial Line | domestic chicken |
| GU261712 | China: Yunnan | domestic chicken |
| GU261709 | India | domestic chicken |
| AY235570 | Commercial Line | domestic chicken |
| AP003580 | Commercial Line | domestic chicken |
| GU261694 | China: Hebei | domestic chicken |
| AP003319 | Laos: Vientiane | domestic chicken |
| HQ857210 | Northeast India | domestic chicken |
| HQ857209 | Northeast India | domestic chicken |
| GU261708 | India | wild fowl |
| HQ857212 | Northeast India | domestic chicken |
| HQ857211 | Northeast India | domestic chicken |
| GU261691 | Myanmar | wild fowl |
| GU261702 | China: Yunnan | wild fowl |
| GU261688 | China: Yunnan | domestic chicken |
| GU261711 | China: Yunnan | domestic chicken |
| GU261689 | China: Yunnan | domestic chicken |
| GU261703 | Myanmar | wild fowl |
| GU261717 | China: Yunnan | domestic chicken |
| DQ648776 | China: Yunnan | domestic chicken |
| GU261678 | China: Henan | domestic chicken |
| GU261710 | China: Yunnan | domestic chicken |
| GU261676 | China: Yunnan | domestic chicken |
| GU261719 | China: Yunnan | domestic chicken |
| GU261690 | China: Yunnan | wild fowl |
| GU261715 | China: Yunnan | domestic chicken |
| GU261706 | China: Yunnan | wild fowl |
| GU261692 | China: Yunnan | wild fowl |
| GU261698 | Northeast India | domestic chicken |
| NC_001323 |  | Commercial Line |
| AP006746 | Japan : Tokyo, Tama Zoological Park | Grey Junglefowl |
| AP003320 | Lao Zoological Park,Vientiane, Lao | Grey Junglefowl |
| AP003321 | Vientiane, Las | Red Jungle Fowl : Spadiceus |
| AP003323 | Indonesia | Red Jungle Fowl : bankiva |
| AP003322 | Philippine | Red Jungle Fowl : gallus |
| AP003325 | Tama Zoological Park, Tokyo, Japan | Ceylon junglefowl(Gallus lafayettei) |
| AP006741 | Delhi National Park, Delhi, India | Grey junglefowl (*Gallus sonnerati*) |
| AP003324 | Bali, Indonesia | Green junglefowl (Gallus varius) |

Table S2. The result summary of Modeltest using 56 models from PAUP.

a) Details of selected model

| Model selected | GTR+I+G |
| --- | --- |
| -lnL | 34137.36 |
| K | 10 |
| AIC | 68294.71 |
| Base frequencies: | |
| freqA = | 0.3043 |
| freqC = | 0.3243 |
| freqG = | 0.1338 |
| freqT = | 0.2377 |
| Substitution model: | |
| Rate matrix | |
| R(a) [A-C] = | 3.1105 |
| R(b) [A-G] = | 69.8681 |
| R(c) [A-T] = | 3.0613 |
| R(d) [C-G] = | 1.2357 |
| R(e) [C-T] = | 55.6961 |
| R(f) [G-T] = | 1 |
| Among-site rate variation | |
| Proportion of invariable sites (I) = | 0.7559 |
| Variable sites (G) | |
| Gamma distribution shape parameter | 1.0295 |

| b) The result of tested 56 models. | | | | | | |
| --- | --- | --- | --- | --- | --- | --- |
| Model | -lnL | K | AIC | delta | weight | cumWeight |
| GTR+I+G | 34137.36 | 10 | 68294.71 | 0 | 0.9751 | 0.9751 |
| TVM+I+G | 34142.09 | 9 | 68302.17 | 7.4609 | 0.0234 | 0.9984 |
| TrN+I+G | 34147.21 | 7 | 68308.43 | 13.7188 | 0.001 | 0.9995 |
| TIM+I+G | 34146.9 | 8 | 68309.8 | 15.0938 | 0.0005 | 1 |
| HKY+I+G | 34152.57 | 6 | 68317.15 | 22.4375 | 1.31E-05 | 1 |
| K81uf+I+G | 34152.25 | 7 | 68318.49 | 23.7812 | 6.68E-06 | 1 |
| GTR+I | 34165.21 | 9 | 68348.41 | 53.7031 | 2.13E-12 | 1 |
| TVM+I | 34171.78 | 8 | 68359.56 | 64.8516 | 8.07E-15 | 1 |
| TrN+I | 34175.44 | 6 | 68362.88 | 68.1719 | 1.53E-15 | 1 |
| TIM+I | 34175.06 | 7 | 68364.12 | 69.4062 | 8.27E-16 | 1 |
| HKY+I | 34182.67 | 5 | 68375.34 | 80.625 | 3.03E-18 | 1 |
| K81uf+I | 34182.28 | 6 | 68376.55 | 81.8438 | 1.65E-18 | 1 |
| GTR+G | 34214.7 | 9 | 68447.39 | 152.6797 | 6.84E-34 | 1 |
| TVM+G | 34218.82 | 8 | 68453.63 | 158.9219 | 3.02E-35 | 1 |
| TrN+G | 34225.78 | 6 | 68463.56 | 168.8516 | 2.11E-37 | 1 |
| TIM+G | 34225.34 | 7 | 68464.69 | 169.9766 | 1.20E-37 | 1 |
| HKY+G | 34230.37 | 5 | 68470.74 | 176.0312 | 5.81E-39 | 1 |
| K81uf+G | 34229.93 | 6 | 68471.86 | 177.1484 | 3.32E-39 | 1 |
| GTR | 34673.59 | 8 | 69363.19 | 1068.477 | 0.00E+00 | 1 |
| TVM | 34676.43 | 7 | 69366.87 | 1072.156 | 0.00E+00 | 1 |
| TrN | 34687.99 | 5 | 69385.98 | 1091.266 | 0.00E+00 | 1 |
| TIM | 34687.3 | 6 | 69386.6 | 1091.891 | 0.00E+00 | 1 |
| HKY | 34690.91 | 4 | 69389.83 | 1095.117 | 0.00E+00 | 1 |
| K81uf | 34690.22 | 5 | 69390.45 | 1095.734 | 0.00E+00 | 1 |
| SYM+I+G | 34994.83 | 7 | 70003.66 | 1708.945 | 0.00E+00 | 1 |
| TVMef+I+G | 35001.57 | 6 | 70015.14 | 1720.43 | 0.00E+00 | 1 |
| SYM+I | 35024.02 | 6 | 70060.04 | 1765.328 | 0.00E+00 | 1 |
| TIMef+I+G | 35029.37 | 5 | 70068.74 | 1774.031 | 0.00E+00 | 1 |
| TVMef+I | 35029.71 | 5 | 70069.42 | 1774.711 | 0.00E+00 | 1 |
| TrNef+I+G | 35031.09 | 4 | 70070.19 | 1775.477 | 0.00E+00 | 1 |
| K81+I+G | 35036.53 | 4 | 70081.06 | 1786.352 | 0.00E+00 | 1 |
| K80+I+G | 35038.25 | 3 | 70082.51 | 1787.797 | 0.00E+00 | 1 |
| TIMef+I | 35058.55 | 4 | 70125.11 | 1830.398 | 0.00E+00 | 1 |
| TrNef+I | 35060.38 | 3 | 70126.76 | 1832.047 | 0.00E+00 | 1 |
| K81+I | 35064.57 | 3 | 70135.13 | 1840.422 | 0.00E+00 | 1 |
| K80+I | 35066.41 | 2 | 70136.82 | 1842.109 | 0.00E+00 | 1 |
| SYM+G | 35064.74 | 6 | 70141.48 | 1846.773 | 0.00E+00 | 1 |
| TVMef+G | 35074.1 | 5 | 70158.2 | 1863.492 | 0.00E+00 | 1 |
| TIMef+G | 35098.94 | 4 | 70205.88 | 1911.164 | 0.00E+00 | 1 |
| TrNef+G | 35100.85 | 3 | 70207.7 | 1912.992 | 0.00E+00 | 1 |
| K81+G | 35108.72 | 3 | 70223.44 | 1928.727 | 0.00E+00 | 1 |
| K80+G | 35110.64 | 2 | 70225.29 | 1930.578 | 0.00E+00 | 1 |
| SYM | 35504.9 | 5 | 71019.8 | 2725.086 | 0.00E+00 | 1 |
| TVMef | 35518.96 | 4 | 71045.92 | 2751.211 | 0.00E+00 | 1 |
| TIMef | 35538.52 | 3 | 71083.05 | 2788.336 | 0.00E+00 | 1 |
| TrNef | 35540.54 | 2 | 71085.07 | 2790.359 | 0.00E+00 | 1 |
| K81 | 35552.56 | 2 | 71109.13 | 2814.414 | 0.00E+00 | 1 |
| K80 | 35554.58 | 1 | 71111.16 | 2816.445 | 0.00E+00 | 1 |
| F81+I+G | 35659.85 | 5 | 71329.7 | 3034.992 | 0.00E+00 | 1 |
| F81+I | 35682.82 | 4 | 71373.64 | 3078.93 | 0.00E+00 | 1 |
| F81+G | 35705.14 | 4 | 71418.27 | 3123.563 | 0.00E+00 | 1 |
| F81 | 36112.66 | 3 | 72231.31 | 3936.602 | 0.00E+00 | 1 |
| JC+I+G | 36487.18 | 2 | 72978.36 | 4683.648 | 0.00E+00 | 1 |
| JC+I | 36510.67 | 1 | 73023.34 | 4728.633 | 0.00E+00 | 1 |
| JC+G | 36530.41 | 1 | 73062.82 | 4768.109 | 0.00E+00 | 1 |
| JC | 36931.09 | 0 | 73862.19 | 5567.477 | 0.00E+00 | 1 |

-lnL : Negative log likelihood; K : Number of estimated parameters; AIC : Akaike Information Criterion; delta : Information difference; weight : Information weight; cumWeight : Cumulative information weight


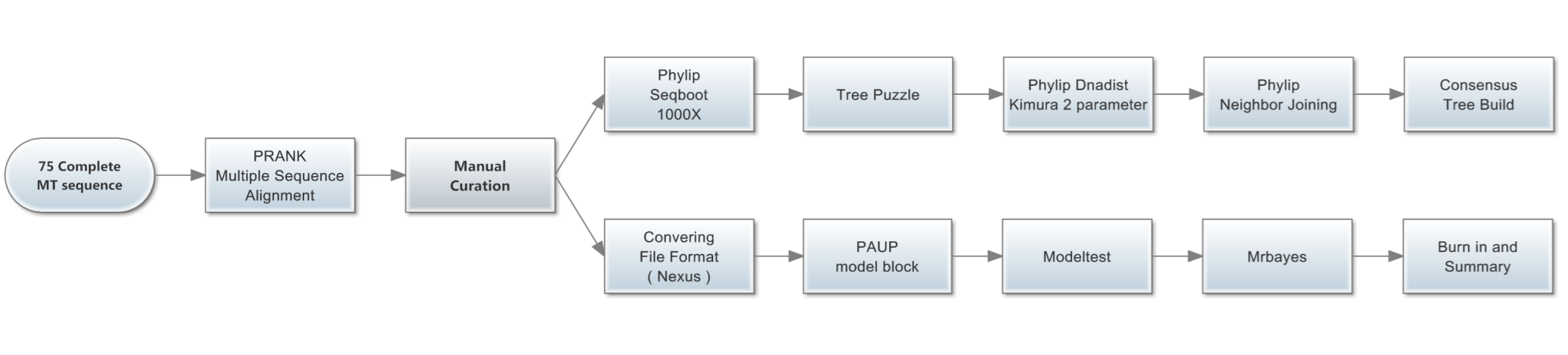


Figure S2. An overview of the phylogenetic analysis process.

Table S3. The result summary of read mapping using Bowtie2

| **Categories** | **Samples** | | | | |
| --- | --- | --- | --- | --- | --- |
|  | **KNC_R(16)** | **KNC_Y(24)** | **KNC_W(3)** | **KNC_L(40)** | **KNC_G(53)** |
| Total number of reads (pairs) | 174,786,737 (100%) | 184,357,557 (100%) | 183,203,458 (100%) | 176,744,880 (100%) | 187,177,967 (100%) |
| Concordantly 1 time (pair) | 153,196,154 (87.65%) | 162,391,840 (88.09%) | 159,423,218 (87.02%) | 152,887,235 (86.50%) | 164,537,465 (87.90%) |
| Concordantly > 1 time (pair) | 10,219,837 (5.85%) | 11,528,236 (6.25%) | 11,388,706 (6.22%) | 11,064,103 (6.26%) | 11,580,493 (6.19%) |
| Discordantly 1 time (pair) | 3,836,219 (33.74%) | 2,977,379 (28.53%) | 4,699,222 (37.92%) | 4,914,884 (38.42%) | 2,173,681 (19.65%) |
| aligned exactly 1 time (singleton) | 4,704,925 (31.22%) | 4,368,759 (29.28%) | 4,677,955 (30.41%) | 4,955,881 (31.45%) | 5,337,267 (30.03%) |
| aligned > 1 time (singleton) | 1,887,982 (12.53%) | 1,797,388 (12.05%) | 2,202,578 (14.32%) | 2,172,806 (13.79%) | 1,831,168 (10.30%) |
| Overall alignment rate | 97.58% | 97.63% | 97.68% | 97.56% | 97.17% |


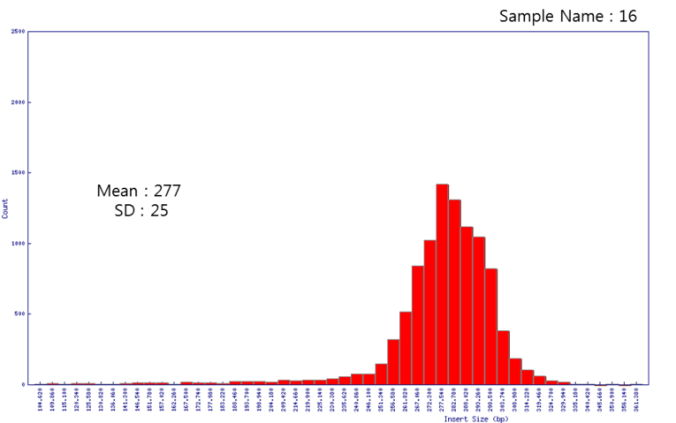

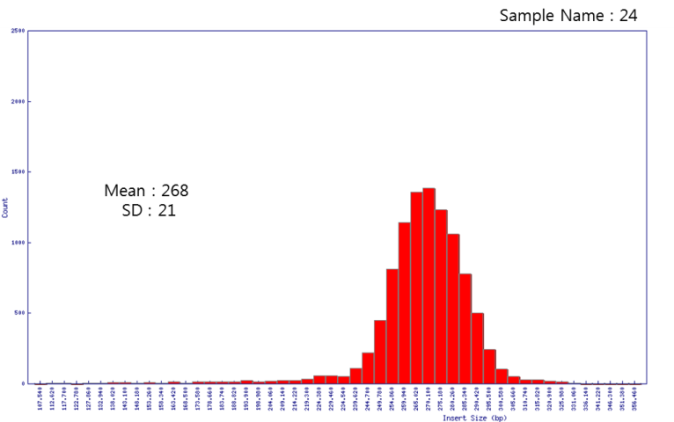

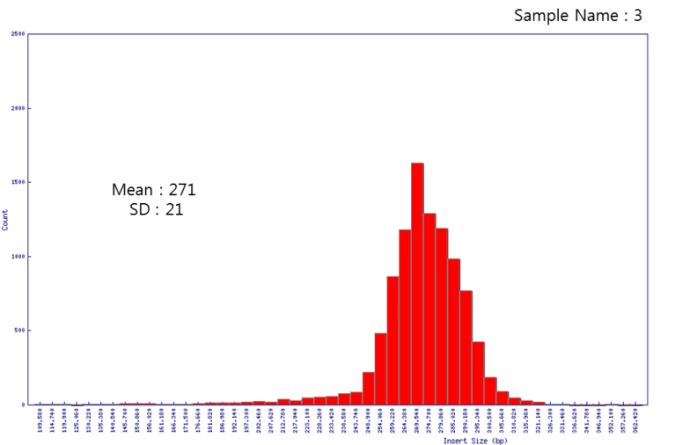

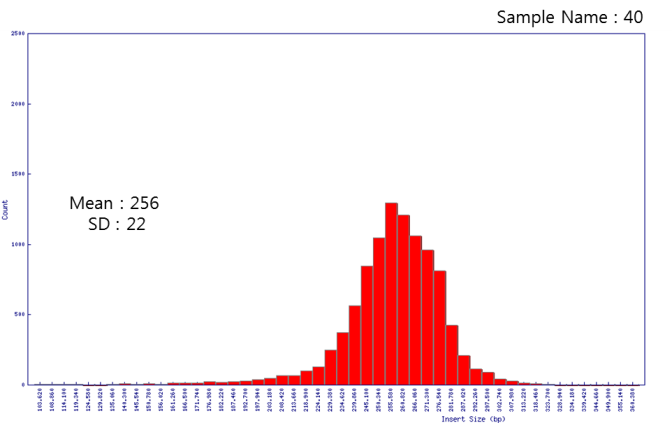

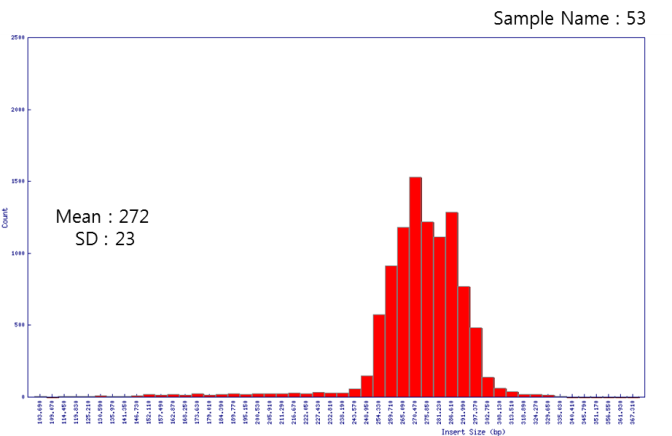


Figure S3. Insert size distributions of 5 KNC samples.

Figure S4. Distribution of known and novel SNVs in each chromosome.

Table S4. The result summary of variant calling using GATK.

| **Categories** | | **Samples** | | | | |
| --- | --- | --- | --- | --- | --- | --- |
|  |  | **KNC_R(16)** | **KNC_Y(24)** | **KNC_W(3)** | **KNC_L(40)** | **KNC_G(53)** |
| **INDEL** | Raw (1,531,995) | 53,172 | 59,314 | 58,898 | 71,510 | 53,302 |
|  | Pass INDEL (1,345,019) | 47,822 | 53,098 | 52,599 | 63,503 | 48,954 |
|  | Missing filter (1,339,266) | 47,471 | 52,723 | 52,262 | 63,035 | 48,481 |
| **SNV** | Raw (12,507,596) | 612,571 | 699,118 | 687,205 | 833,459 | 635,840 |
|  | Pass SNP (11,063,897) | 550,343 | 631,217 | 619,550 | 755,175 | 571,380 |
|  | Missing filter (10,962,086) | 545,252 | 626,348 | 615,689 | 749,082 | 565,418 |

* The number in sample column of INDEL & SNP : the number of sample specific variants


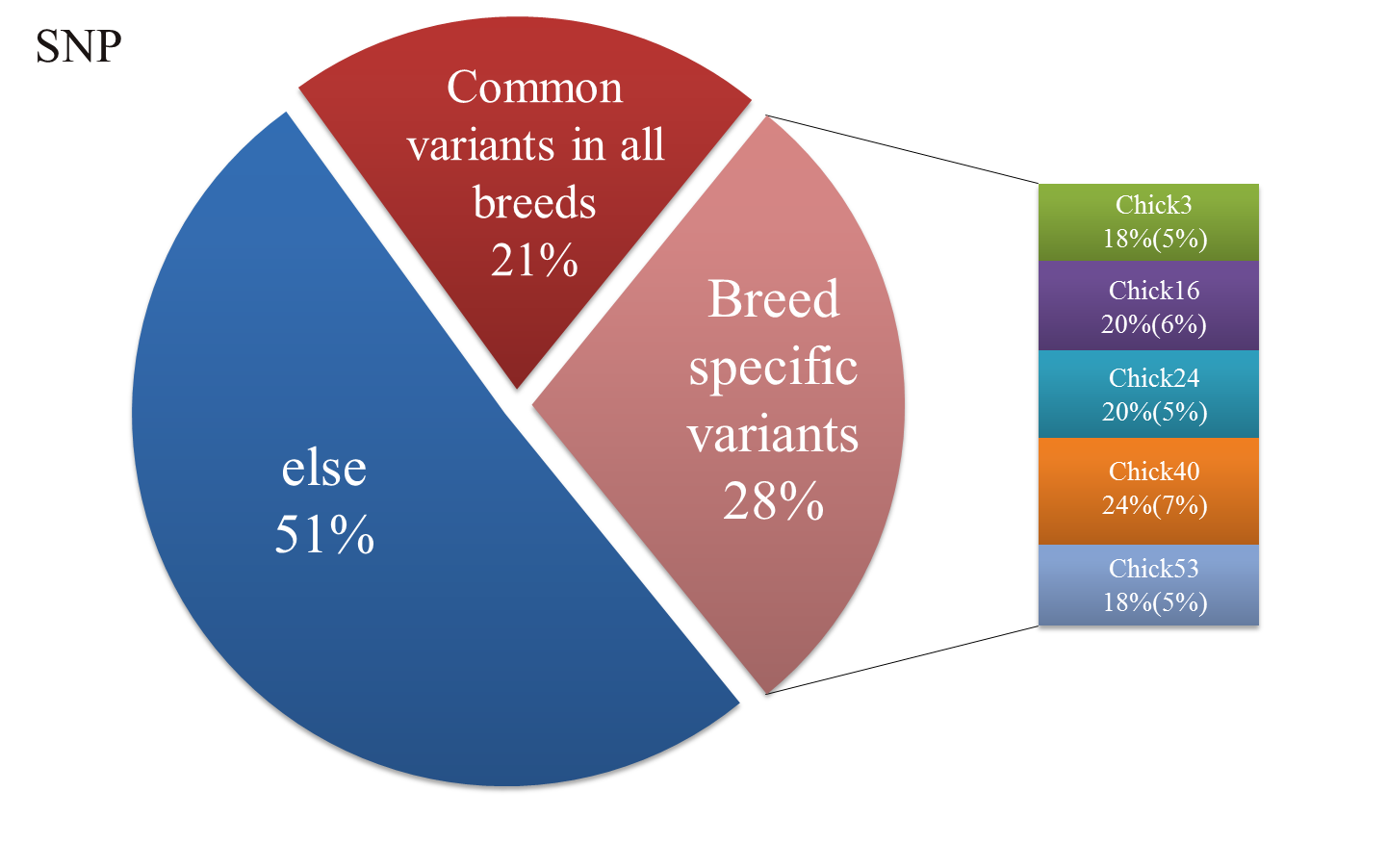


Figure S5. Ratio of breed specific SNV and the proportion of each breed in breed specific SNVs.


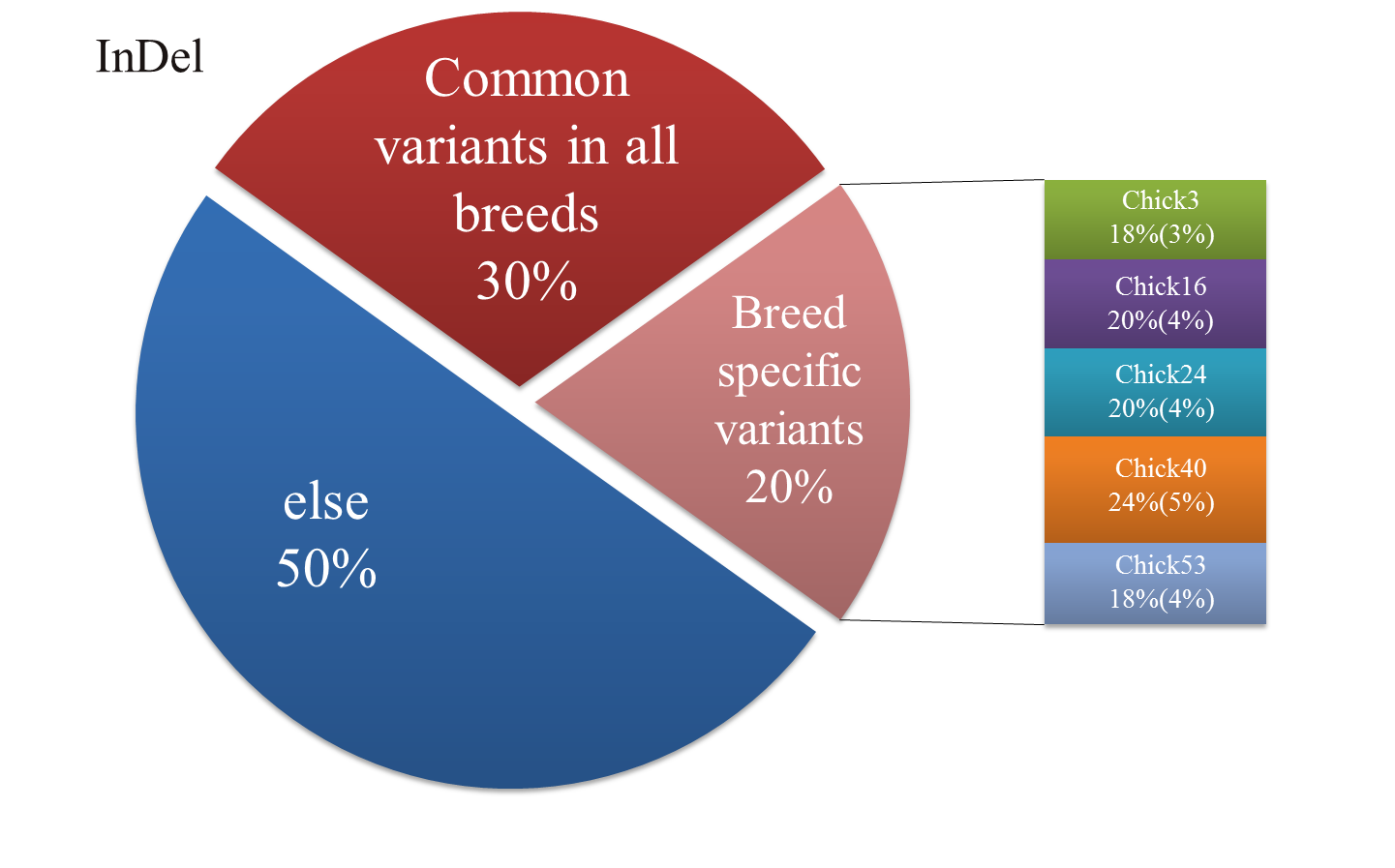


Figure S6. Ratio of breed specific InDel and the proportion of each breed in breed specific InDels.

Figure S7. Ratio of the number of chromosomal variants to the total number of variant.

**
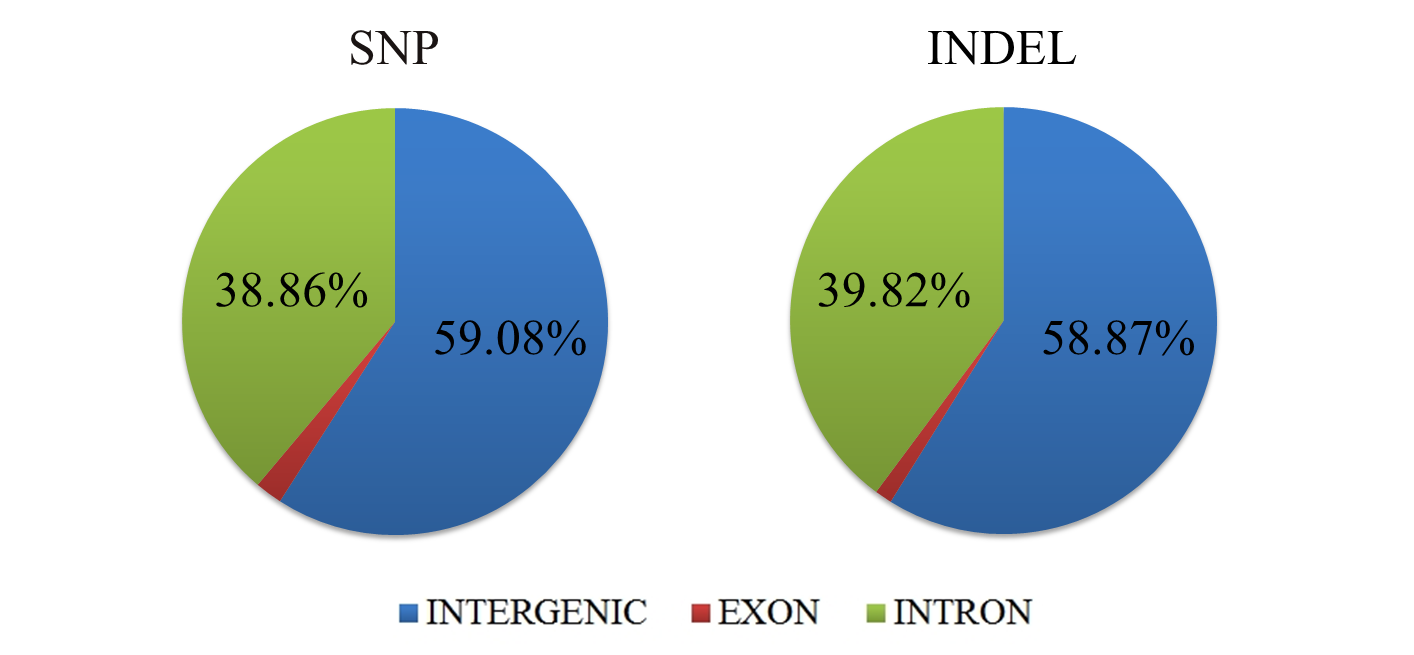
**

Figure S8. Ratio of each variant location in three variant types (SNP, InDel and CNV).

Table S5. The list of synonymous and non-synonymous variants in common genes of highly variable regions.

| Chr | Position | rs ID | Gene Name | Ref | Alt | Variant Type |
| --- | --- | --- | --- | --- | --- | --- |
| 6 | 4224056 | . | PCDH15 | C | T | Synonymous |
| 6 | 4229110 | rs313675060 | PCDH15 | G | A | Non-synonymous |
| 6 | 4229173 | rs13564223 | PCDH15 | T | C | Synonymous |
| 6 | 4229224 | rs312847192 | PCDH15 | C | T | Synonymous |
| 6 | 4232521 | rs313099267 | PCDH15 | T | C | Synonymous |
| 6 | 4232554 | rs14568172 | PCDH15 | A | G | Synonymous |
| 6 | 4234475 | . | PCDH15 | G | A | Synonymous |
| 6 | 4236298 | rs314575975 | PCDH15 | G | A | Synonymous |
| 6 | 4236376 | rs313725350 | PCDH15 | A | G | Non-synonymous |
| 6 | 4241555 | rs313104422 | ENSGALG00000006716 | C | T | Non-synonymous |
| 6 | 4241620 | . | ENSGALG00000002638 | G | A | Non-synonymous |
| 6 | 4241659 | . | ENSGALG00000002638 | G | A | Non-synonymous |
| 6 | 4241660 | rs316029067 | ENSGALG00000002638 | T | C | Synonymous |
| 6 | 4253773 | rs312946121 | CCDC61 | C | T | Synonymous |
| 6 | 4255187 | . | CCDC61 | C | T | Synonymous |
| 6 | 4261940 | rs316995093 | CCDC61 | C | T | Synonymous |
| 6 | 4261985 | . | CCDC61 | C | A | Synonymous |
| 6 | 4262012 | rs314660677 | CCDC61 | T | A | Synonymous |
| 6 | 4262051 | . | CCDC61 | G | A | Synonymous |
| 6 | 4285266 | rs15767865 | PCDH15 | A | G | Synonymous |
| 6 | 4287469 | rs16535684 | PCDH15 | C | T | Synonymous |
| 6 | 4290453 | rs315541806 | PCDH15 | A | G | Synonymous |
| 6 | 4290504 | rs317404631 | PCDH15 | T | C | Synonymous |
| 6 | 4296263 | rs317485464 | PCDH15 | C | T | Synonymous |
| 6 | 4301893 | rs317750367 | PCDH15 | A | G | Synonymous |
| 6 | 4303282 | rs318016312 | PCDH15 | G | T | Synonymous |
| 6 | 4303357 | rs317241809 | PCDH15 | G | A | Synonymous |
| 6 | 4305072 | rs317951418 | PCDH15 | C | T | Synonymous |
| 6 | 4308645 | rs312273510 | PCDH15 | G | A | Synonymous |
| 6 | 4308665 | rs312744667 | PCDH15 | G | A | Synonymous |
| 6 | 4310205 | . | PCDH15 | C | T | Non-synonymous |
| 6 | 4310346 | . | PCDH15 | T | A | Synonymous |
| 6 | 4310349 | rs313345140 | PCDH15 | A | C | Non-synonymous |
| 6 | 4310424 | rs313539303 | PCDH15 | T | A | Synonymous |
| 6 | 4315980 | rs14568074 | PCDH15 | G | A | Synonymous |
| 6 | 4321280 | rs13564171 | PCDH15 | C | T | Synonymous |
| 6 | 4550555 | rs313275476 | BMS1 | T | G | Non-synonymous |
| 6 | 4550736 | rs16532257 | BMS1 | G | T | Synonymous |
| 6 | 4551519 | rs317329335 | BMS1 | T | C | Synonymous |
| 6 | 4551558 | rs316986733 | BMS1 | C | G | Synonymous |
| 6 | 4552075 | rs315693216 | BMS1 | T | C | Synonymous |
| 6 | 4552111 | rs16532261 | BMS1 | C | T | Synonymous |
| 6 | 4556162 | rs15762384 | BMS1 | C | T | Non-synonymous |
| 6 | 4556163 | rs15762386 | BMS1 | T | C | Synonymous |
| 6 | 4556190 | rs15762388 | BMS1 | G | T | Non-synonymous |
| 6 | 4556202 | rs315518222 | BMS1 | T | C | Synonymous |
| 6 | 4556939 | . | BMS1 | A | G | Synonymous |
| 6 | 4558644 | rs315416387 | BMS1 | A | C | Non-synonymous |
| 6 | 4560802 | rs316213482 | BMS1 | A | G | Synonymous |
| 6 | 4560847 | rs317188690 | BMS1 | T | C | Synonymous |
| 6 | 4561476 | rs316786682 | BMS1 | G | A | Synonymous |
| 6 | 4562567 | rs314849160 | BMS1 | T | C | Synonymous |
| 6 | 4562990 | rs317079533 | BMS1 | T | C | Non-synonymous |
| 6 | 4562996 | . | BMS1 | G | T | Non-synonymous |
| 6 | 4563001 | rs317476030 | BMS1 | A | G | Non-synonymous |
| 6 | 4563039 | rs316639983 | BMS1 | C | T | Synonymous |
| 6 | 4563082 | . | BMS1 | T | C | Non-synonymous |
| 6 | 4563132 | rs315205899 | BMS1 | C | T | Synonymous |
| 6 | 4563260 | rs14564917 | BMS1 | G | C | Non-synonymous |
| 6 | 4563263 | rs14564918 | BMS1 | T | G | Non-synonymous |
| 6 | 4563327 | rs313735300 | BMS1 | A | G | Synonymous |
| 6 | 4563534 | rs315188932 | BMS1 | A | G | Synonymous |
| 6 | 4563684 | rs314859638 | BMS1 | T | G | Non-synonymous |
| 6 | 4563694 | . | BMS1 | A | G | Non-synonymous |
| 6 | 4565768 | . | BMS1 | T | A | Synonymous |
| 6 | 4567342 | . | BMS1 | C | T | Synonymous |
| 6 | 4568712 | . | BMS1 | A | G | Synonymous |
| 6 | 4568747 | . | BMS1 | C | T | Non-synonymous |
| 6 | 4569862 | rs16532272 | BMS1 | T | A | Synonymous |
| 6 | 4569868 | rs313312360 | BMS1 | G | A | Synonymous |
| 6 | 4569931 | rs313756588 | BMS1 | G | C | Synonymous |
| 6 | 4569934 | rs314668522 | BMS1 | G | A | Synonymous |
| 6 | 4594840 | rs15762493 | ENSGALG00000002638 | C | T | Synonymous |
| 6 | 4594846 | rs15762495 | ENSGALG00000002638 | C | T | Synonymous |
| 6 | 4602082 | rs314875596 | ENSGALG00000002638 | G | C | Synonymous |
| 6 | 4602427 | rs13563020 | ENSGALG00000002638 | T | C | Synonymous |
| 6 | 4602482 | rs13563021 | ENSGALG00000002638 | G | A | Synonymous |
| 6 | 4602554 | rs15762565 | ENSGALG00000002638 | G | A | Synonymous |
| 6 | 4603807 | rs313870152 | ENSGALG00000002638 | A | G | Synonymous |
| 6 | 4603839 | rs317650965 | ECD | G | A | Synonymous |
| 6 | 4604756 | rs313075079 | ECD | T | C | Synonymous |
| 6 | 4605603 | . | ECD | A | G | Non-synonymous |
| 6 | 4606787 | rs14565165 | ECD | T | C | Non-synonymous |
| 6 | 4606821 | . | ECD | G | A | Synonymous |
| 6 | 4606875 | . | ECD | C | G | Non-synonymous |
| 6 | 4611381 | rs14565175 | ECD | A | C | Synonymous |
| 6 | 4612274 | . | ECD | C | A | Non-synonymous |
| 6 | 4612298 | . | DNAJC9 | C | T | Synonymous |
| 6 | 4614558 | rs316313079 | DNAJC9 | C | T | Synonymous |
| 6 | 4614605 | rs315862237 | DNAJC9 | G | C | Synonymous |
| 6 | 4640356 | . | ENSGALG00000002638 | A | G | Synonymous |
| 6 | 4640682 | . | ENSGALG00000002638 | T | G | Non-synonymous |
| 6 | 4640763 | rs317776059 | ENSGALG00000002638 | G | A | Synonymous |
| 6 | 4640778 | rs13563007 | ENSGALG00000002638 | A | G | Synonymous |
| 6 | 4644587 | rs315130613 | ANXA11 | C | T | Synonymous |
| 6 | 4647572 | . | ANXA11 | G | T | Non-synonymous |
| 6 | 4649167 | rs29008631 | ANXA11 | T | C | Non-synonymous |
| 6 | 4650390 | . | ANXA11 | G | A | Synonymous |
| 6 | 4650414 | . | ANXA11 | T | G | Synonymous |
| 6 | 4650423 | rs10728385 | ANXA11 | C | T | Synonymous |
| 6 | 4654557 | . | ANXA11 | G | A | Non-synonymous |
| 6 | 4670889 | rs314713975 | DNAJC9 | T | C | Synonymous |
| 6 | 4671092 | rs315236461 | DNAJC9 | A | G | Synonymous |
| 6 | 4671139 | rs314907519 | DNAJC9 | T | C | Synonymous |
| 6 | 4671496 | rs15762794 | DNAJC9 | A | C | Synonymous |
| 6 | 4672430 | rs315606443 | CSGALNACT2 | A | T | Synonymous |
| 6 | 4672439 | . | CSGALNACT2 | A | G | Non-synonymous |
| 6 | 4674376 | rs16531676 | CSGALNACT2 | C | G | Synonymous |
| 6 | 4675902 | rs16531677 | CSGALNACT2 | A | G | Synonymous |
| 6 | 4678603 | rs312355735 | ENSGALG00000027731 | A | G | Synonymous |
| 6 | 4678621 | . | ENSGALG00000024441 | G | A | Synonymous |
| 6 | 4678690 | rs314294621 | ENSGALG00000024441 | A | G | Synonymous |
| 6 | 4678696 | rs14565098 | ENSGALG00000024441 | G | A | Synonymous |
| 6 | 4678729 | rs14565099 | ENSGALG00000024441 | T | C | Synonymous |
| 6 | 4679275 | . | ENSGALG00000024315 | T | C | Non-synonymous |
| 6 | 4680578 | . | ENSGALG00000024315 | C | A | Synonymous |
| 6 | 4680640 | . | ENSGALG00000024315 | G | C | Non-synonymous |
| 6 | 4680644 | . | ENSGALG00000024315 | G | A | Non-synonymous |
| 6 | 4681212 | rs13767528 | ENSGALG00000006724 | C | T | Non-synonymous |
| 6 | 4682906 | rs314902080 | ENSGALG00000006724 | G | A | Synonymous |
| 6 | 4682967 | . | ENSGALG00000006724 | C | T | Non-synonymous |
| 6 | 4682973 | . | ENSGALG00000006724 | T | C | Synonymous |
| 6 | 4683006 | . | ENSGALG00000006724 | C | T | Non-synonymous |
| 6 | 4683355 | rs314474478 | ENSGALG00000006724 | C | T | Synonymous |
| 6 | 4684560 | rs316894803 | ENSGALG00000006724 | T | C | Synonymous |
| 6 | 4685253 | rs312620330 | ENSGALG00000006724 | T | C | Synonymous |
| 6 | 4685265 | . | ENSGALG00000006724 | C | T | Synonymous |
| 6 | 4685319 | rs312967217 | ENSGALG00000006716 | G | A | Non-synonymous |
| 6 | 4685414 | . | ENSGALG00000006716 | C | T | Non-synonymous |
| 6 | 4687735 | rs312329987 | ENSGALG00000006716 | G | A | Non-synonymous |
| 6 | 4687756 | . | ENSGALG00000006716 | T | G | Non-synonymous |
| 6 | 4688261 | . | ENSGALG00000006716 | T | C | Non-synonymous |
| 6 | 4689061 | rs317498266 | ENSGALG00000006716 | A | G | Synonymous |
| 6 | 4689096 | rs313950362 | ENSGALG00000006716 | G | A | Non-synonymous |
| 6 | 4692890 | . | CSGALNACT2 | C | T | Synonymous |
| 6 | 4693714 | rs16531678 | CSGALNACT2 | T | C | Synonymous |
| 6 | 4693723 | rs314727659 | CCDC61 | A | G | Synonymous |
| 6 | 4693765 | rs317164084 | CCDC61 | C | T | Synonymous |
| 6 | 4693882 | rs315310730 | CCDC61 | C | T | Synonymous |
| 6 | 4693900 | rs315798135 | CCDC61 | G | A | Non-synonymous |
| 6 | 4694398 | rs15966534 | CCDC61 | T | C | Synonymous |
| 6 | 4698453 | rs317462158 | PCDH15 | C | T | Synonymous |
| 6 | 4698457 | rs314707952 | PCDH15 | A | G | Synonymous |
| 6 | 4698553 | rs315247411 | PCDH15 | G | A | Non-synonymous |
| 6 | 4699774 | rs312740292 | PCDH15 | T | C | Synonymous |
| 6 | 4705668 | rs318156073 | PCDH15 | T | C | Synonymous |
| 6 | 4706005 | rs313790177 | PCDH15 | G | A | Synonymous |
| 6 | 4751043 | . | FAM149B1 | C | G | Non-synonymous |
| 6 | 4751071 | . | FAM149B1 | C | T | Non-synonymous |
| 6 | 4756618 | . | FAM149B1 | C | G | Non-synonymous |
| 6 | 4756646 | rs14565206 | FAM149B1 | C | T | Synonymous |
| 6 | 4756676 | rs13563100 | FAM149B1 | C | T | Synonymous |
| 6 | 4756712 | rs312913804 | FAM149B1 | C | A | Synonymous |
| 6 | 4760333 | . | FAM149B1 | T | G | Non-synonymous |
| 6 | 4761416 | . | FAM149B1 | G | A | Non-synonymous |
| 6 | 4761476 | . | FAM149B1 | G | A | Synonymous |
| 6 | 4761605 | . | ENSGALG00000027731 | C | T | Synonymous |
| 6 | 4761686 | . | ENSGALG00000027731 | C | T | Non-synonymous |
| 6 | 4761866 | . | ENSGALG00000027731 | C | T | Non-synonymous |
| 6 | 5484685 | rs314355527 | ENSGALG00000002638 | C | T | Non-synonymous |
| 6 | 5484747 | rs312307453 | ENSGALG00000002638 | G | A | Synonymous |
| 6 | 5484773 | rs315183890 | ENSGALG00000002638 | G | A | Synonymous |
| 6 | 5484778 | rs15762491 | ENSGALG00000002638 | G | A | Synonymous |
| 6 | 5764889 | rs15768437 | PCDH15 | G | A | Synonymous |
| 6 | 5841405 | rs317478407 | PCDH15 | T | C | Synonymous |
| 6 | 5849337 | rs314167769 | PCDH15 | C | G | Non-synonymous |
| 6 | 5854828 | rs314658012 | PCDH15 | T | C | Synonymous |
| 6 | 5854885 | . | IPMK | C | T | Synonymous |
| 6 | 5855954 | rs14565379 | IPMK | A | C | Synonymous |
| 6 | 5896370 | rs15763074 | IPMK | C | T | Non-synonymous |
| 6 | 5896405 | . | IPMK | T | C | Synonymous |
| 6 | 5907970 | rs316024078 | IPMK | A | T | Synonymous |
| 6 | 5908054 | . | IPMK | A | T | Synonymous |
| 6 | 5927714 | rs313732930 | IPMK | T | C | Non-synonymous |
| 6 | 5931579 | rs314831555 | IPMK | C | T | Synonymous |
| 6 | 5955268 | rs315540873 | IPMK | A | C | Synonymous |
| 6 | 5955331 | rs315478119 | IPMK | C | T | Synonymous |
| 6 | 5955427 | rs316390358 | IPMK | A | G | Synonymous |
| 6 | 5955538 | . | IPMK | T | G | Non-synonymous |
| 6 | 5955840 | rs317769899 | IMP4 | C | T | Synonymous |
| 6 | 5955960 | . | IMP4 | G | A | Synonymous |
| 6 | 5969998 | . | IMP4 | T | A | Synonymous |
| 6 | 5970001 | . | IMP4 | A | G | Synonymous |
| 6 | 5970015 | . | IMP4 | A | G | Synonymous |
| 6 | 5970058 | rs10729093 | IMP4 | T | G | Synonymous |
| 6 | 5980813 | . | IMP4 | G | A | Non-synonymous |
| 6 | 5980832 | rs316895297 | FAM149B1 | C | T | Synonymous |
| 6 | 5983013 | rs316146231 | FAM149B1 | A | G | Synonymous |
| 6 | 5994263 | rs14565179 | FAM149B1 | C | A | Synonymous |
| 6 | 6003140 | rs317678981 | FAM149B1 | C | T | Synonymous |
| 6 | 6018558 | rs14565180 | FAM149B1 | T | C | Synonymous |
| 6 | 6034967 | . | FAM149B1 | C | T | Synonymous |
| 6 | 6035039 | rs314702035 | FAM149B1 | C | T | Synonymous |
| 6 | 6035096 | rs313751671 | FAM149B1 | C | T | Non-synonymous |
| 6 | 6039802 | rs13563079 | FAM149B1 | A | G | Synonymous |
| 6 | 6039844 | rs15762736 | FAM149B1 | G | A | Synonymous |
| 6 | 6039904 | rs313587760 | FAM149B1 | T | C | Non-synonymous |
| 6 | 6042928 | rs13563088 | FAM149B1 | C | T | Synonymous |
| 6 | 6057204 | rs316567292 | FAM149B1 | C | T | Synonymous |
| 6 | 6062644 | . | FAM149B1 | A | G | Synonymous |
| 6 | 6062739 | rs312370989 | FAM149B1 | C | T | Non-synonymous |
| 6 | 6062887 | . | FAM149B1 | C | T | Synonymous |
| 9 | 2487445 | . | RET | G | C | Synonymous |
| 9 | 2577715 | rs314421041 | RET | G | A | Synonymous |
| 9 | 2730278 | . | RET | T | C | Synonymous |
| 9 | 2730327 | rs16531716 | RET | T | C | Synonymous |
| 9 | 2778003 | . | RET | G | T | Synonymous |
| 9 | 2778133 | rs316945363 | RET | G | T | Synonymous |
| 9 | 2778262 | rs315231537 | RET | G | A | Synonymous |
| 9 | 2891405 | rs16531737 | RET | A | G | Synonymous |
| 9 | 2891411 | . | RET | T | C | Synonymous |
| 9 | 2934998 | rs13563116 | UBE2D1 | G | A | Synonymous |
| 9 | 2936265 | rs313427622 | UBE2D1 | T | A | Synonymous |
| 9 | 2937083 | rs314014023 | TFAM | T | G | Synonymous |
| 9 | 2937143 | rs316175688 | TFAM | T | A | Synonymous |
| 9 | 2939530 | . | TFAM | G | A | Synonymous |
| 9 | 2939539 | . | TFAM | C | T | Non-synonymous |
| 9 | 2939611 | rs16531695 | RET | G | A | Synonymous |
| 9 | 2941477 | . | RASGEF1A | C | T | Synonymous |
| 9 | 2941660 | rs312271878 | RASGEF1A | T | C | Synonymous |
| 9 | 2941967 | rs313102556 | RASGEF1A | C | T | Synonymous |
| 9 | 2942207 | rs313063862 | RASGEF1A | T | C | Synonymous |
| 9 | 2942702 | . | RASGEF1A | C | T | Synonymous |
| 9 | 2942723 | . | RASGEF1A | G | A | Synonymous |
| 9 | 2942762 | . | RASGEF1A | G | A | Synonymous |
| 9 | 2942941 | rs313421108 | RASGEF1A | C | T | Synonymous |
| 9 | 2942944 | rs14567666 | PCDH15 | C | T | Synonymous |
| 9 | 2943447 | rs318080911 | PCDH15 | A | G | Synonymous |
| 9 | 2943905 | . | PCDH15 | A | G | Non-synonymous |
| 9 | 2947606 | rs16531739 | RET | T | C | Non-synonymous |
| 9 | 2947653 | rs316593153 | RET | G | A | Synonymous |
| 9 | 2947752 | rs316965365 | RET | G | A | Synonymous |
| 9 | 2947764 | rs315633520 | RET | G | A | Synonymous |
| 9 | 2947771 | rs16531742 | RET | G | A | Synonymous |
| 9 | 2947896 | . | RET | C | A | Synonymous |
| 9 | 2947925 | rs317667870 | RET | A | T | Non-synonymous |
| 9 | 2948312 | rs316508568 | RASGEF1A | C | T | Synonymous |
| 9 | 2964587 | rs315474274 | VPS8 | C | T | Synonymous |
| 9 | 2964659 | . | VPS8 | G | A | Synonymous |
| 9 | 2964661 | rs313638194 | VPS8 | T | G | Non-synonymous |
| 9 | 2964689 | . | VPS8 | G | A | Synonymous |
| 9 | 2964703 | . | VPS8 | C | G | Non-synonymous |
| 9 | 2966730 | . | VPS8 | G | A | Synonymous |
| 9 | 2968065 | rs317770278 | VPS8 | C | T | Synonymous |
| 9 | 2978724 | rs313718066 | VPS8 | T | C | Synonymous |
| 9 | 2978784 | rs312489261 | VPS8 | G | A | Synonymous |
| 9 | 2978802 | rs312612149 | VPS8 | C | T | Synonymous |
| 9 | 2986533 | rs316723108 | VPS8 | T | A | Synonymous |
| 9 | 2987211 | rs317044479 | VPS8 | C | T | Synonymous |
| 9 | 2987229 | rs15966449 | VPS8 | C | T | Synonymous |
| 9 | 2987885 | rs318002862 | VPS8 | T | C | Synonymous |
| 9 | 2988225 | . | VPS8 | C | T | Synonymous |
| 9 | 2992770 | . | VPS8 | G | A | Synonymous |
| 9 | 2998087 | rs313002505 | VPS8 | G | A | Synonymous |
| 9 | 2998117 | rs316667358 | VPS8 | A | G | Synonymous |
| 9 | 2999688 | . | VPS8 | C | T | Synonymous |
| 9 | 3000738 | rs13767093 | VPS8 | A | G | Synonymous |
| 9 | 3008682 | . | VPS8 | C | T | Synonymous |
| 9 | 3008742 | rs15966354 | VPS8 | G | A | Synonymous |
| 9 | 3010720 | rs313634050 | VPS8 | G | A | Non-synonymous |
| 9 | 3011787 | . | VPS8 | C | T | Synonymous |
| 9 | 3019944 | rs15966298 | VPS8 | G | A | Synonymous |
| 9 | 3019954 | . | VPS8 | A | G | Non-synonymous |
| 9 | 3020953 | rs14670233 | VPS8 | T | C | Synonymous |
| 9 | 3029811 | . | VPS8 | T | A | Non-synonymous |
| 9 | 3029824 | rs314031788 | VPS8 | T | C | Synonymous |
| 9 | 3029833 | rs317753651 | VPS8 | C | T | Synonymous |

Table S6. The result summary of Korean Native Chicken genome assembly using IDBA_UD.

| **Sample name** | **KNC_R(16)** | **KNC_Y(24)** | **KNC_W(3)** | **KNC_L(40)** | **KNC_G(53)** |
| --- | --- | --- | --- | --- | --- |
| **Number of contig** | 60,290 | 62,213 | 58,616 | 61,911 | 57,044 |
| **Sequence length** |  |  |  |  |  |
| Minimum length | 2,000 | 2,000 | 2,000 | 2,000 | 2,000 |
| Maximum length | 283,645 | 314,996 | 491,386 | 389,965 | 350,576 |
| Average length | 15,800 | 15,308 | 16,385 | 15,453 | 16,882 |
| N50 length | 26,987 | 25,437 | 27,910 | 25,694 | 29,018 |
| **Residue information** |  |  |  |  |  |
| Total residue count(bp) | 952,589,481 | 952,359,183 | 960,479,414 | 956,749,716 | 963,026,148 |
| N contents | 58,428 | 35,411 | 43,377 | 30,066 | 43,123 |
| Closed N by Gapcloser | 99,173 | 71,852 | 82,573 | 40,460 | 80,508 |
| GC content (%) | 40.91% | 40.90% | 41.08% | 41.01% | 41.14% |

Table S7. The result summary of remapping unaligned reads to assembled genome using bowtie2.

| **Categories** | **Samples** | | | | |
| --- | --- | --- | --- | --- | --- |
|  | **KNC_R(16)** | **KNC_Y(24)** | **KNC_W(3)** | **KNC_L(40)** | **KNC_G(53)** |
| Total number of reads | 11,370,743 | 10,437,470 | 12,391,507 | 12,793,558 | 11,060,004 |
| Concordantly 1 time | 1,932,064 (16.99%) | 2,117,448 (20.29%) | 2,066,499 (16.68%) | 1,942,966 (15.19%) | 2,286,405 (20.67%) |
| Concordantly > 1 time | 749,669 (6.59%) | 933,047 (8.94%) | 877,468 (7.08%) | 826,398 (6.46%) | 1,017,384 (9.20%) |
| Discordantly 1 time | 64,955 (0.75%) | 55,537 (0.75%) | 57,547 (0.61%) | 62,611 (0.62%) | 79,465 (1.02%) |
| Overall alignment rate | 24.16% | 29.76% | 24.22% | 22.14% | 30.59% |


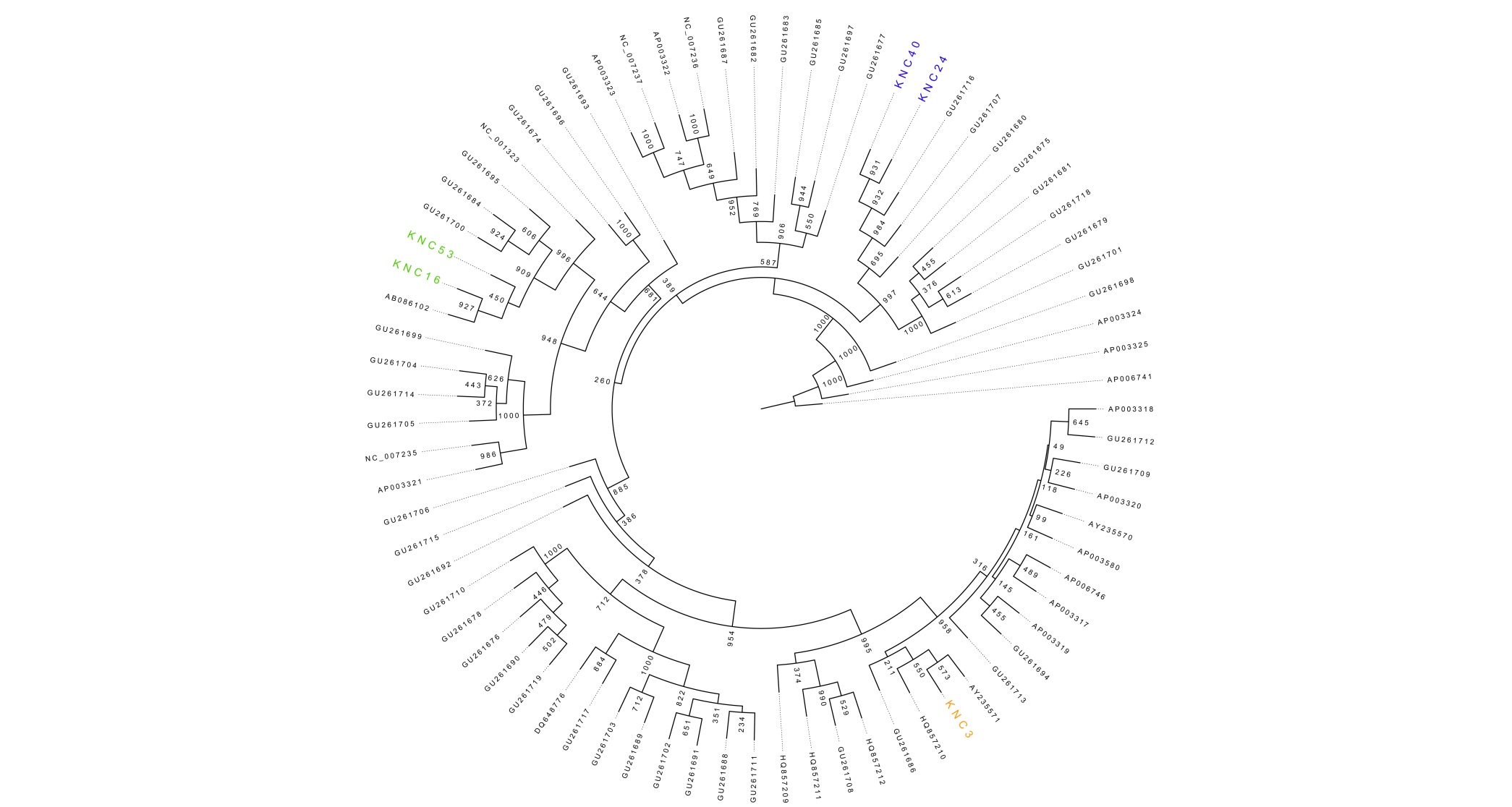


Figure S9. Neighbor joining tree of 75 samples used in this analysis.
